# Collaborative Action of Brca1 and CtIP in Elimination of Covalent Modifications from Double-Strand Breaks to Facilitate Subsequent Break Repair

**DOI:** 10.1371/journal.pgen.1000828

**Published:** 2010-01-22

**Authors:** Kyoko Nakamura, Toshiaki Kogame, Hiroyuki Oshiumi, Akira Shinohara, Yoshiki Sumitomo, Keli Agama, Yves Pommier, Kimiko M. Tsutsui, Ken Tsutsui, Edgar Hartsuiker, Tomoo Ogi, Shunichi Takeda, Yoshihito Taniguchi

**Affiliations:** 1 Department of Radiation Genetics, Graduate School of Medicine, Kyoto University, Kyoto, Japan; 2 Institute for Protein Research, Graduate School of Science, Osaka University, Suita, Osaka, Japan; 3 Laboratory of Molecular Pharmacology, National Cancer Institute, National Institutes of Health, Bethesda, Maryland, United States of America; 4 Department of Neurogenomics, Department of Genome Dynamics, Graduate School of Medicine, Dentistry, and Pharmaceutical Sciences, Okayama University, Okayama, Japan; 5 Cancer Biology, North West Cancer Research Fund Institute, Bangor University, Bangor, United Kingdom; 6 Department of Molecular Medicine, Atomic Bomb Disease Institute, Nagasaki University, Nagasaki, Japan; Brandeis University, United States of America

## Abstract

Topoisomerase inhibitors such as camptothecin and etoposide are used as anti-cancer drugs and induce double-strand breaks (DSBs) in genomic DNA in cycling cells. These DSBs are often covalently bound with polypeptides at the 3′ and 5′ ends. Such modifications must be eliminated before DSB repair can take place, but it remains elusive which nucleases are involved in this process. Previous studies show that CtIP plays a critical role in the generation of 3′ single-strand overhang at “clean” DSBs, thus initiating homologous recombination (HR)–dependent DSB repair. To analyze the function of CtIP in detail, we conditionally disrupted the CtIP gene in the chicken DT40 cell line. We found that CtIP is essential for cellular proliferation as well as for the formation of 3′ single-strand overhang, similar to what is observed in DT40 cells deficient in the Mre11/Rad50/Nbs1 complex. We also generated DT40 cell line harboring CtIP with an alanine substitution at residue Ser332, which is required for interaction with BRCA1. Although the resulting *CtIP^S332A/−/−^* cells exhibited accumulation of RPA and Rad51 upon DNA damage, and were proficient in HR, they showed a marked hypersensitivity to camptothecin and etoposide in comparison with *CtIP^+/−/−^* cells. Finally, *CtIP^S332A/−/−^BRCA1^−/−^* and *CtIP^+/−/−^BRCA1^−/−^* showed similar sensitivities to these reagents. Taken together, our data indicate that, in addition to its function in HR, CtIP plays a role in cellular tolerance to topoisomerase inhibitors. We propose that the BRCA1-CtIP complex plays a role in the nuclease-mediated elimination of oligonucleotides covalently bound to polypeptides from DSBs, thereby facilitating subsequent DSB repair.

## Author Summary

Induction of double-strand breaks (DSBs) in chromosomal DNA effectively activates a program of cellular suicide and is widely used for chemotherapy on malignant cancer cells. Cells resist such therapies by quickly repairing the DSBs. Repair is carried out by two major DSB repair pathways, homologous recombination (HR) and nonhomologous end-joining. However, these pathways cannot join DSBs if their ends are chemically modified, as seen in the DSB ends that would arise after the prolonged treatment of the cells with topoisomerase inhibitors such as camptothecin and etoposide. These anti-cancer drugs can produce the polypeptides covalently attached to the 3′ or 5′ end of DSBs. It remains elusive which enzymes eliminate these chemical modifications prior to repair. We here show evidence that the BRCA1-CtIP complex plays a role in eliminating this chemical modification, thereby facilitating subsequent DSB repair. Thus, BRCA1 and CtIP have dual functions: their previously documented roles in HR and this newly identified function. This study contributes to our ability to predict the effectiveness of chemotherapeutic agents prior to their selection by evaluating the activity of individual repair factors. Accurate prediction is crucial, because chemotherapeutic agents that cause DNA damage have such strong side effects.

## Introduction

CtIP was isolated as a binding partner of CtBP (C-terminal binding protein), and has subsequently been shown to interact with a number of molecules, including BRCA1 (Breast Cancer Susceptibility Gene 1) [1]. *CtIP* is a functional homolog of yeast S*ae2* (Sporulation in the Absence of Spo Eleven), and acts at the initial step of homologous recombination (HR)-dependent double-strand break (DSB) repair [2],[3]. HR is initiated by forming 3′ single-strand (ss) overhangs at DSBs. In this resection step, Sae2/CtIP works together with a complex composed of Mre11/Rad50/Xrs2 in budding yeast, or with Mre11/Rad50/Nbs1 in mammals [4]–[7]. The Rad51 recombinase protein polymerizes on the ss DNA overhang, and the resulting ssDNA-Rad51 complex undergoes homology search. Resection activity is upregulated by phosphorylation of a conserved residue in Sae2 by the cyclin-dependent kinase (CDK) [8]. This phosphorylation site is conserved in human CtIP (Thr847), and is also phosphorylated by CDK [7].

BRCA1 was originally identified as a tumor suppressor gene associated with familial breast and ovarian cancer [9]. BRCA1 contains an N-terminal RING-finger domain, and is associated with structurally related BARD1 to form an E3-ubiquitin ligase. BRCA1/BARD1 forms three distinct complexes with Abraxas, Bach1 and CtIP, and plays a role in DNA repair [10]. BRCA1 binds to CtIP in a manner that is dependent on the phosphorylation of CtIP at Ser327 [11],[12]. Following DNA damage, the ubiquitylation of CtIP by BRCA1 causes the migration of CtIP towards a chromatin fraction [12]. However, the biological significance of the complex formed between BRCA1 and CtIP has not yet been clarified.

Topoisomerases 1 and 2 (Topo1 and Topo2) have been drawing increasing attention as important targets for cancer therapy, since the inhibition of these enzymes causes DSBs during DNA replication [13]. Topo1 and Topo2 induce single-strand breaks (SSBs) and DSBs, respectively. Covalent bonds are transiently formed between Topo1 and the 3′ end of the SSB and between Topo2 and the 5′ end of the DSB [14]. The anti-cancer agent camptothecin (CPT) inhibits Topo1 by stabilizing the Topo1-cleavage complex, which interferes with replication, and thereby induces DSBs in one of the sister chromatids [15]. Topo2 inhibitors such as etoposide (VP16) and ICRF-193 also kill cycling cells and are used in cancer therapy. VP16 stabilizes the Topo2-cleavage complex, while ICRF-193 stabilizes the closed clamp which forms after the strand passage [16],[17]. Topo1-mediated DNA damage caused by CPT is repaired primarily by homologous recombination (HR), while Topo2-mediated DNA damage caused by VP16 or ICRF-193 is mainly repaired by nonhomologous end joining (NHEJ) [18],[19]. It should be noted that the repair of CPT- and VP16-induced DSBs requires an additional step: the elimination of covalently bound polypeptides from the DNA ends. Hartsuiker et al. demonstrated that Topo2 is removed from DNA by the collaborative action of the MRX complex and ctp1 (the ortholog of CtIP) in fission yeast [20]. It remains to be seen whether vertebrate CtIP shares the same function as in yeast, which does not have BRCA1 ortholog.

To understand the role of the BRCA1-CtIP interaction, we substituted the Ser332 residue (equivalent to human Ser327) of CtIP with alanine in the chicken DT40 B lymphocyte line [21],[22]. In addition, to analyze the function of CtIP, we conditionally depleted *CtIP* in DT40 cells. We here show that the depletion of CtIP is lethal to cells as is the inactivation of Mre11, Rad50, and Nbs1 [23],[24], indicating the critical role played by CtIP in HR. Remarkably, although the CtIP S332A mutation had no significant impact on HR, it made cells hypersensitive to CPT and VP16 but not to ICRF193. These observations unmasked an unexpected function of the BRCA1-CtIP interaction: cellular tolerance to the DSBs that are covalently associated with the polypeptides. Our data therefore support two distinct functions of CtIP: the resection of DSBs in HR and the elimination of polypeptides from the cleavage complex.

## Results

### CtIP is required for the assembly of Rad51 at DNA damage sites

**Figure 1:**
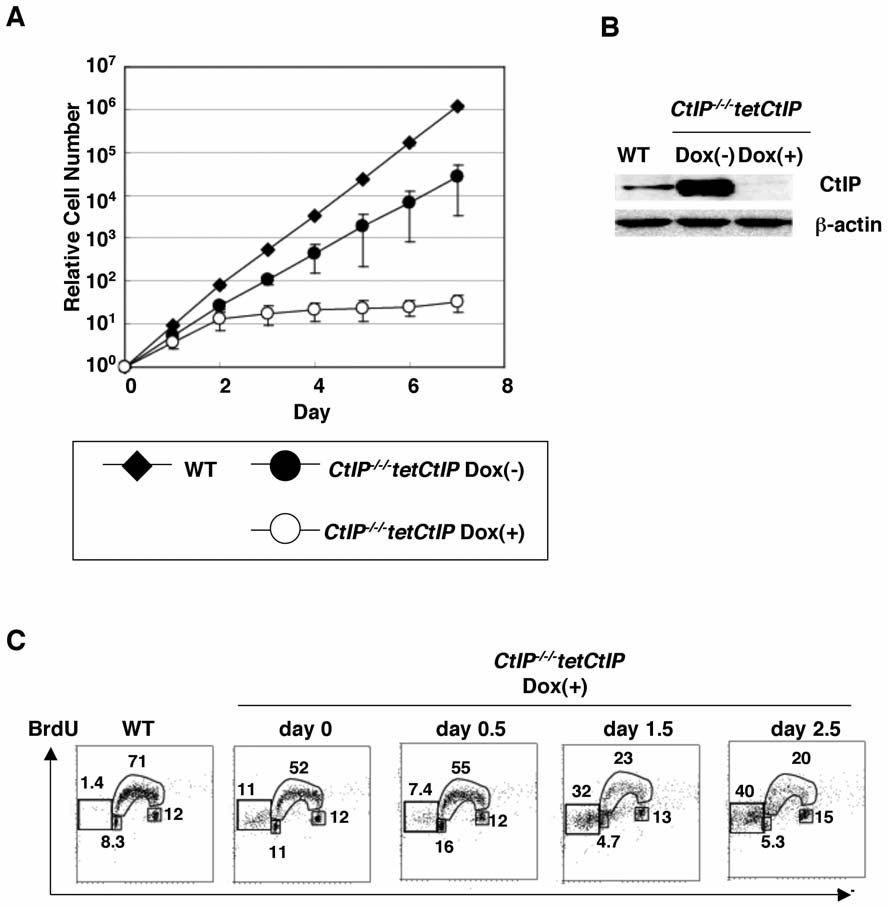
CtIP is essential for cell survival. (A) Defective proliferation of *CtIP^−/−/−^tetCtIP* cells following addition of doxycycline (2 µg/ml) to deplete CtIP, from time zero. Data shown are the average results from three separate clones. Points indicate mean (n = 3); bars indicate S.D. (B) Western blot analysis of chicken CtIP expression. Whole cell lysates were prepared from *CtIP^−/−/−^tetCtIP* cells 24 h after addition of doxycycline. β-actin was used as a loading control. (C) Cell cycle distribution of *CtIP^−/−/−^tetCtIP* cells at the indicated time after addition of doxycycline. Cells were pulse-labeled with BrdU for 10 min and subsequently stained with FITC-conjugated anti-BrdU antibody (Y axis, log scale) and propidium iodide (PI) (X axis, linear scale). Each square on the left-hand side represents apoptic cells (sub-G_1_ fraction). The small rectangle at the bottom of the tubular arch, the tubular arch, and the small square to the right represent cells in the G_1_, S, and G_2_/M phases, respectively.

In order to determine the function of CtIP, we conditionally disrupted the *CtIP* gene in chicken DT40 cells, using a chicken *CtIP* transgene under the control of a tetracycline-repressible promoter (*tetCtIP* transgene, Figure S1A). We designed *CtIP* gene-disruption constructs, so that the amino acid sequences from 96 to 335 would be replaced by selection-marker genes. Since the gene is encoded on chromosome 2, which is in trisomy in DT40, we disrupted three *CtIP* alleles (Figure S1B and S1C). The resulting *CtIP^−/−/−^tetCtIP* cells tended to grow more slowly than did *wild-type* cells, presumably due to overexpression of the *tetCtIP* transgene (Figure 1A and 1B). To deplete the *CtIP* in the *CtIP^−/−/−^tetCtIP* cells, we added doxycycline (modified tetracycline) to the culture medium. One day after the addition of doxycycline, the amount of CtIP was reduced to around 20% of *wild-type* cells (Figure 1B), and the cells started dying as evidenced by an increase in the sub-G_1_ fraction (Figure 1C). This lethality can be attributed to abolished HR, because the cells showed a significant increase in the number of spontaneous chromosomal breaks (Table 1), as do *Mre11*- and *Rad51*-depleted cells [23],[25]. By day 3, the vast majority of the *CtIP^−/−/−^tetCtIP* cells had stopped growing and died (Figure 1A and 1C). We therefore conclude that CtIP is essential for maintenance of chromosomal DNA and cellular proliferation.

**Table 1:** Chromosomal aberrations in *CtIP^−/−/−^tetCtIP* mutants.

	Genotype	Chromatid-type	Chromosome-type
IR 0 Gy	*Wild-type*	6	9
	*CtIP^−/−/−^tetCtIP*Dox(−)	4	13
	*CtIP^−/−/−^tetCtIP*Dox(+)	7	28
IR 2 Gy	*Wild-type*	10	24
	*CtIP^−/−/−^tetCtIP*Dox(−)	6	25
	*CtIP^−/−/−^tetCtIP*Dox(+)	20	71

γ-ray irradiated (2 Gy) and non-irradiated cells were treated with colcemid for 3 h to enrich mitotic cells prior to fixation of cells for chromosome analysis. Data are presented as the number of aberrations per 100 cells.

**Figure 2:**
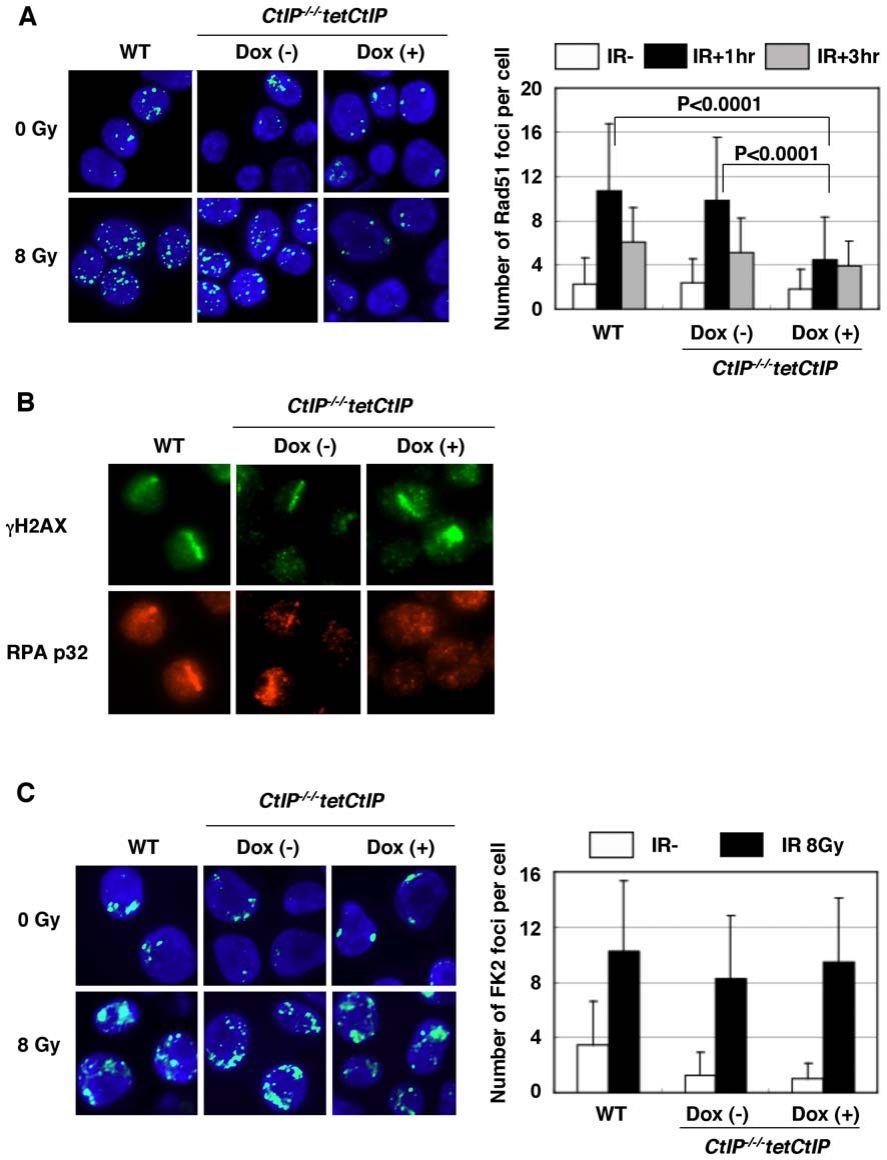
Impaired γ-ray–induced Rad51 focus formation in *CtIP*-depleted cells. (A) *CtIP^−/−/−^tetCtIP* cells, with or without 24 h doxycycline treatment, were exposed to 8 Gy ionizing radiation (IR). One hour after irradiation, cells were stained with anti-Rad51 antibody. The graph shows the quantification of Rad51 foci per cell. More than 100 cells were analyzed. (B) Accumulation of RPA protein in the cells exposed to 405 nm pulse laser. Cells were fixed with 4% PFA 1 h post laser stimulation and stained with anti-γH2AX and anti-RPA^p32^ antibodies. (C) Conjugated-ubiquitin (FK2) focus formation in *wild-type* (WT) or *CtIP^−/−/−^tetCtIP* DT40 cells (with or without doxycycline) at 1 h post IR (8Gy). Quantification of FK2 foci per cell is shown in the graph. More than 100 cells were analyzed.

To assess the HR capability of *CtIP^−/−/−^tetCtIP* cells, we monitored the recruitment of Rad51 and RPA to DNA damage sites one day after addition of doxycycline. Clear Rad51 foci appeared in *wild-type* cells one hour after ionizing radiation (IR), whereas Rad51 foci were hardly detectable in the *CtIP*-depleted cells (Figure 2A). Likewise, the depletion of *CtIP* abolished the accumulation of RPA on DNA lesions induced by microlaser treatment (Figure 2B). This is consistent with a phenotype shown in the previous report [3]. Thus, CtIP plays an essential role in the resection of DSBs during HR in DT40 cells as well as in mammalian cells.

We next investigated whether or not CtIP facilitates the activation of BRCA1 at DSBs. To this end, we measured the formation of conjugated-ubiquitin foci at DSBs, since Brca1 promotes extensive ubiquitylation at IR-induced DSBs [26]. Previous studies showed that *BRCA1^−/−^* DT40 cells exhibit a prominent defect in the formation of conjugated-ubiquitin foci [27]. In contrast, *CtIP* depletion did not reduce the ubiquitylation of DNA damage sites (Figure 2C), suggesting that CtIP is not required for the activation of Brca1.

### Proficient HR in *CtIP^S332A/−/−^* DT40 clones

**Figure 3:**
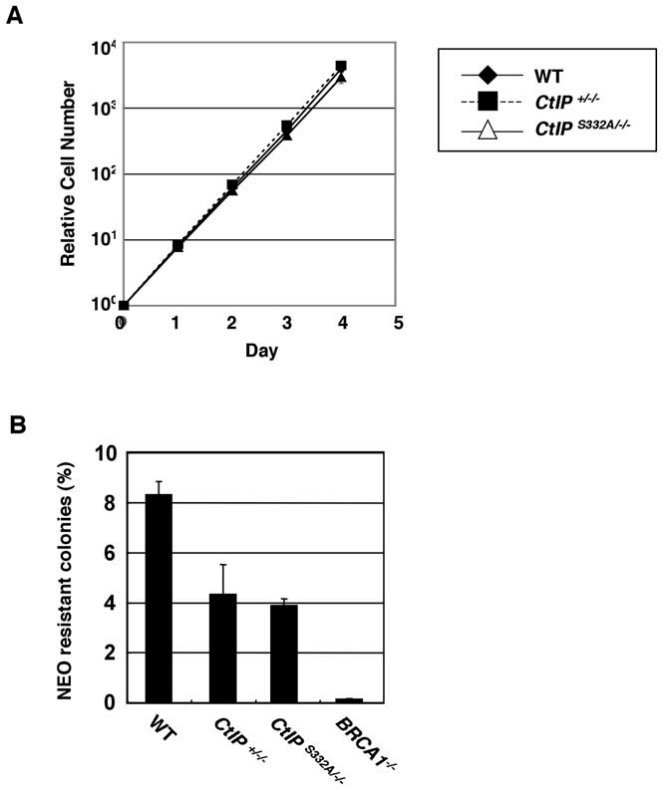
Proficient homologous recombination in *CtIP^S332A/−/−^* clones. (A) Growth kinetics of the indicated CtIP mutant clones. (B) I-*Sce*1–induced DSBs stimulate homologous recombination (HR) in an artificial substrate, *SCneo*. The recombination frequency was calculated by dividing the number of neomycin-resistant colonies by the number of the total colonies. Error bars indicate S.D.

To functionally analyze the interaction of CtIP with BRCA1, we generated *CtIP^S332A/−/−^* cells, in which the critical amino acid in the binding interface has been mutated (Figure S2A, S2B, S2C). The *CtIP^S332A/−/−^* DT40 clones were capable of proliferating at a rate similar to the *CtIP^+/−/−^* cells without a prominent change in the cell-cycle profile (Figure 3A and Figure S2D). Western blot analysis showed that the S332A CtIP proteins were expressed at the similar level to the *wild-type* protein, indicating that amino acid substitutions do not affect the stability of the CtIP protein (Figure S2E). As expected, given the results of a previous study [12], the S332A mutation of CtIP indeed inhibited its interaction with BRCA1 (Figure S2F).

To evaluate the capability of HR in *CtIP^S332A/−/−^* cells, we integrated an artificial substrate, *SCneo*, in the *Ovalbumin* locus [28], and measured the efficiency of I-*Sce*I-induced gene conversion. The *CtIP^S332A/−/−^* clones showed no significant decrease in the appearance of neomycin-resistant colonies compared to *CtIP^+/−/−^* cells (Figure 3B). The proficient HR in *CtIP^S332A/−/−^* DT40 clones is in marked contrast to the severe phenotype of the *Nbs1^p70^* hypomorphic mutant, which exhibited a 10-fold reduction of the gene-targeting frequency and a 10^3^-fold decrease in the efficiency of HR in the *SCneo* substrate [29]. Next, we measured the frequency of gene targeting at the *CENP-H* and *Ovalbumin* loci. In contrast to I-*Sce*I-induced gene conversion, the gene-targeting frequency of the *CtIP^S332A/−/−^* clones decreased moderately in comparison with *CtIP^+/−/−^* cells (Table 2). We speculate that this is because unknown recombination intermediates that require processing by CtIP/BRCA1 may arise during gene targeting event (see Discussion).

**Table 2:** Targeted integration frequencies of *CtIP^S332A/−/−^* clones.

	Targeted loci	
Genotype	*CENP-H*	*Ovalbumin*
*Wild-type*	38/61 (62.2%)	24/26 (92.3%)
*CtIP^+/−/−^*	42/64 (65.6%)	25/30 (83.3%)
*CtIP^S332A/−/−^*	25/71 (35.2%)	30/47 (63.8%)
*BRCA1* ^−/−^	N.D.	5/37 (13.5%)

Gene-targeting efficiencies were determined by flow cytometry for *CENP-H* locus and by Southern blot analysis for *Ovalbumin* locus. N.D. indicates not determined.

**Figure 4:**
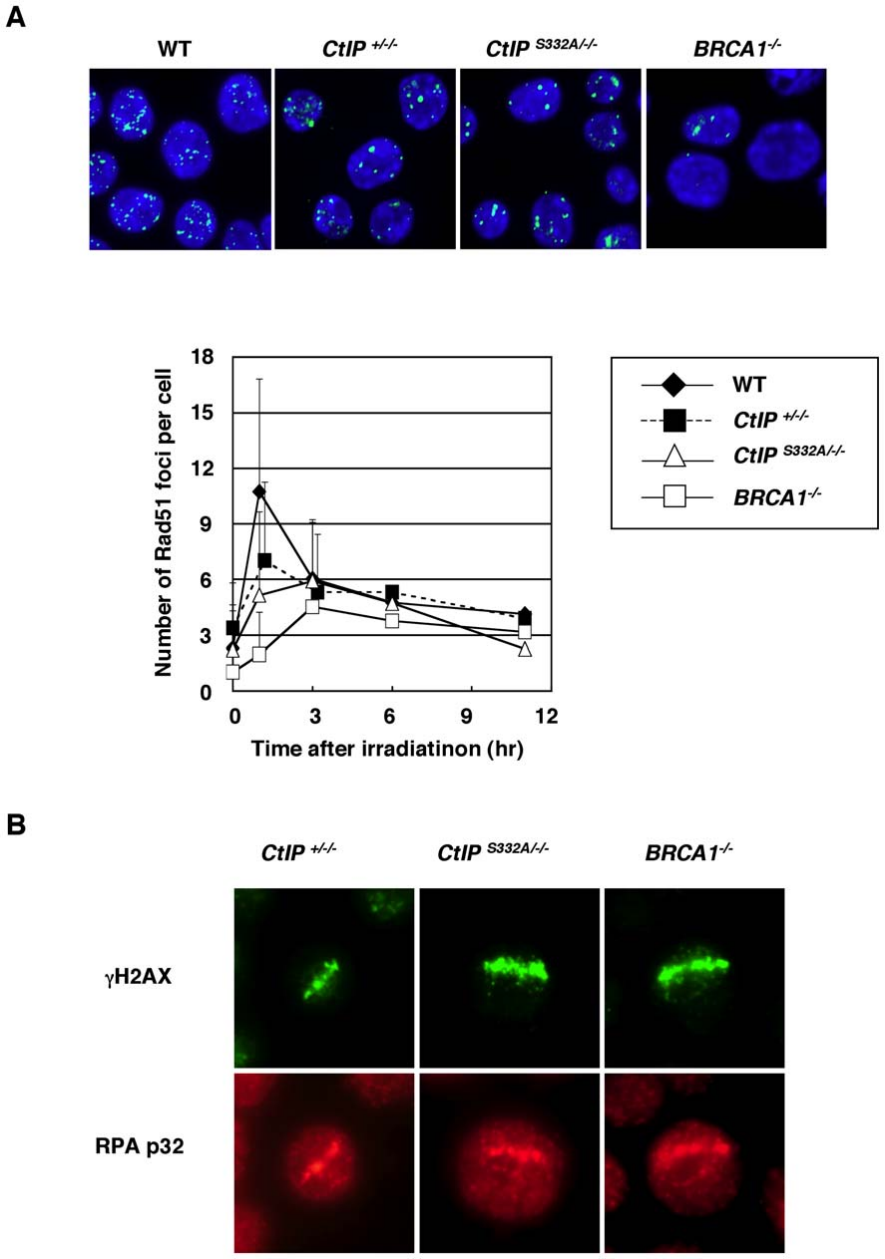
Accumulation of Rad51 and RPA at DNA damage sites is indistinguishable between *CtIP^+/−/−^* and *CtIP^S332A/−/−^* clones. (A) Rad51 focus formation in CtIP mutant cells. The cells of indicated genotype were irradiated with 8Gy of γ-ray, and were immuno-stained with anti-Rad51 antibodies 1 h after irradiation. The lower graph shows the quantification of Rad51 foci per cell. More than 100 cells were analyzed. Error bars indicate S.D. (B) The subnuclear accumulation of RPA in the cells exposed to 405 nm pulse laser. The cells were fixed with 4% PFA 1 h after exposure, and were stained with anti-γH2AX and anti-RPA^p32^ antibodies.

Fluorescent immunostaining revealed that the kinetics of Rad51 focus formation after γ-irradiation was indistinguishable between *CtIP^S332A/−/−^* cells and the *CtIP^+/−/−^* control cells, while *BRCA1^−/−^* cells showed the significant reduction in the Rad51 focus formation at 1–6 h after irradiation (Figure 4A). Furthermore, the *CtIP^S332A/−/−^* mutants displayed laser-induced RPA accumulation as did the *CtIP^+/−/−^*cells (Figure 4B). Laser-generated RPA accumulation following BrdU incorporation largely arises from the resection rather than other routes of single strand formation such as the damage caused by laser itself or replication-associated single strand formation, because RPA accumulation is abolished specifically in *Ubc13* deficient cells [27]. This suggests that *CtIP^S332A/−/−^* cells are proficient in resection at DSB sites. Taken together, we conclude that the S332A mutation of CtIP does not significantly compromise HR.

### *CtIP^S332A/−/−^* cells display a marked hypersensitivity to both CPT and VP16, which stabilize the Topo-cleavage complexes

**Figure 5:**
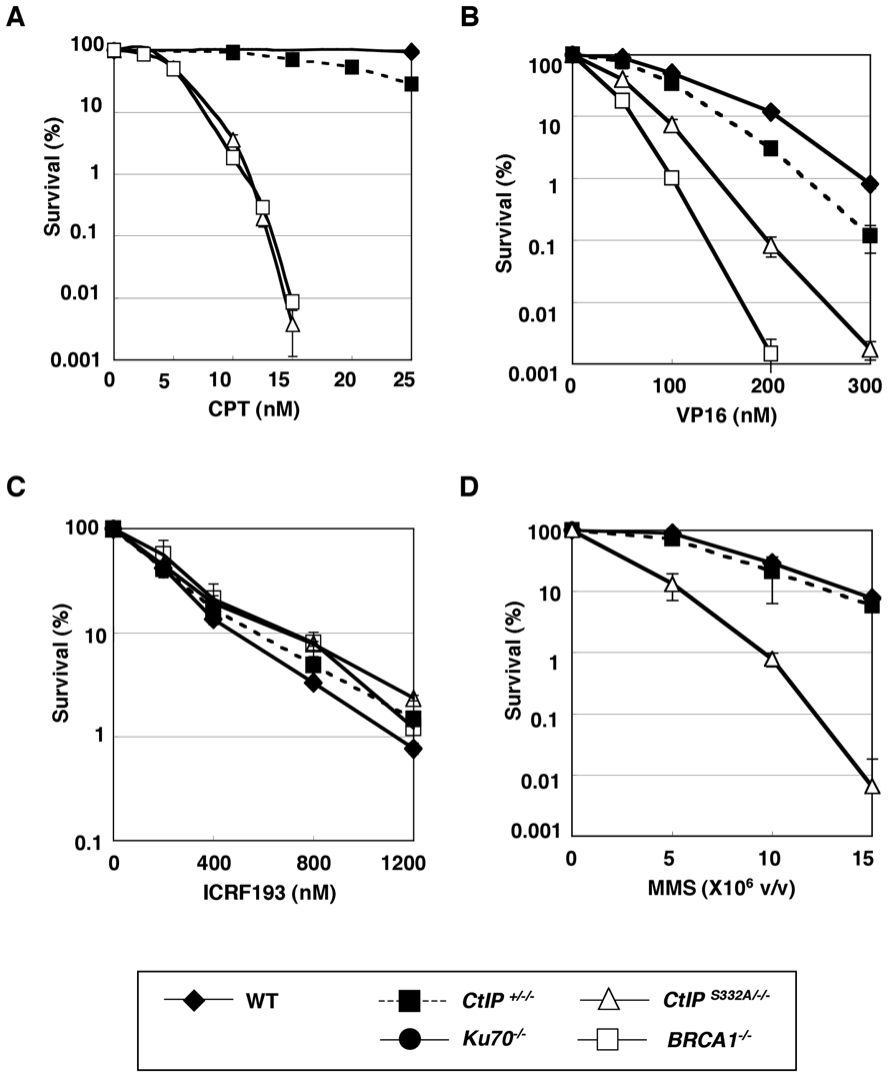
Colony survival assay of *CtIP* mutant cells to various genotoxic agents. Colony survival assay of asynchronous populations of cells exposed to (A) camptothecin (CPT), (B) etoposide (VP16), (C) ICRF-193, and (D) methyl methanesulfonate (MMS). The dose is displayed on the X axis on a linear scale, while the percent fraction of surviving colonies is displayed on the Y axis on a logarithmic scale. To count the surviving colonies, cells were grown for 8–10 days in methylcellulose medium containing various concentrations of DNA–damaging agents. At least three independent experiments were performed with triplicate samples, and the representative results are shown. The error bars indicate S.D.

To determine the role of CtIP in the cellular response to DNA damage, we measured the sensitivity of the *CtIP* mutant cells to various genotoxic agents using a colony survival assay. *CtIP^+/−/−^* cells exhibited the slightly elevated sensitivity toward CPT and VP16 (Figure 5A and 5B), though they expressed the similar level of CtIP protein to the *wild-type* cells (Figure S2E). It is possible that the difference in the amount of CtIP protein between *CtIP^+/−/−^* and *wild-type* cells is too subtle to detect, and that even the suboptimal level of CtIP protein renders the cells sensitive to genotoxic stimuli. A compensatory post-translational regulation may be present because *CtIP^+/−/−^* cells exhibited about 80% reductions in *CtIP* mRNA level compared to the *wild-type* level (Figure S2G). In contrast to *CtIP^+/−/−^* cells, *CtIP^S332A/−/^* ^−^ mutants showed a significantly increased sensitivity to VP16 and MMS (Figure 5B and 5D), but not to γ-rays (data not shown). Furthermore, the sensitivity to CPT was dramatically elevated in the *CtIP^S332A/−/^* ^−^ mutants, in comparison with the *CtIP^+/−/−^* cells (Figure 5A).

The contribution of CtIP to the cellular tolerance to VP16 indicated that CtIP might play a role in NHEJ [18]. To test this hypothesis, we evaluated NHEJ by measuring the sensitivity of *CtIP* mutant cells to ICRF-193, because ICRF193-induced DNA lesions are repaired exclusively by NHEJ, whereas a fraction of the VP16-induced DSBs are repaired by HR [18]. The *CtIP^S332A/−/^* ^−^ clones exhibited no increased ICRF193 sensitivity (Figure 5C). NHEJ can also be evaluated by measuring the IR sensitivity of the cell population at the G_1_ phase, where NHEJ plays a dominant role in DSB repair [30]. The *CtIP* hypomorphic mutants synchronized at the G_1_ phase did not show significant IR hypersensitivity (Figure S3). These observations indicate that NHEJ is not impaired in *CtIP^S332A/−/^* ^−^ clones. In summary, in comparison with *CtIP^+/−/−^* cells, *CtIP^S332A/−/^* ^−^ clones exhibited a significantly higher sensitivity to CPT and VP16, although these clones exhibited no decrease in the efficiency of HR or NHEJ. We conclude that CtIP can therefore contribute to cellular tolerance to CPT and VP16, independently of HR or NHEJ, most likely by eliminating covalently bound polypeptides from the DSBs.

### Epistatic relationship of CtIP to BRCA1 in cellular tolerance to CPT and VP16

CtIP physically interacts with BRCA1 in a manner dependent on phosphorylation of Ser332 [12]. In order to assess the functional relationship between Ser332 phosphorylation of CtIP and BRCA1, we disrupted the *BRCA1* gene in the *CtIP^S332A/−/−^* and *CtIP^+/−/−^* clones (Figure S4A and S4B), as was done previously [31]. Both the *CtIP^S332A/−/−^BRCA1^−/−^* and the *CtIP^+/−/−^BRCA1^−/−^* clones proliferated with similar rates at significantly reduced growth rates, in comparison with *BRCA1^−/−^*cells (doubling time ± SD: 8.3±0.2 h for *wild-type*, 9.3±0.3 h for *BRCA1^−/−^*, 11±0.3 h for *CtIP^+/−/−^BRCA1^−/−^*, 12.6±0.9 h for *CtIP^S332A/−/−^BRCA1^−/−^*). The viability of *CtIP^S332A/−/−^BRCA1^−/−^* cells is in marked contrast with the lethality of *CtIP*-null cells, supporting the idea that the CtIP-BRCA1 interaction works independently from the function of CtIP in resection.

**Figure 6:**
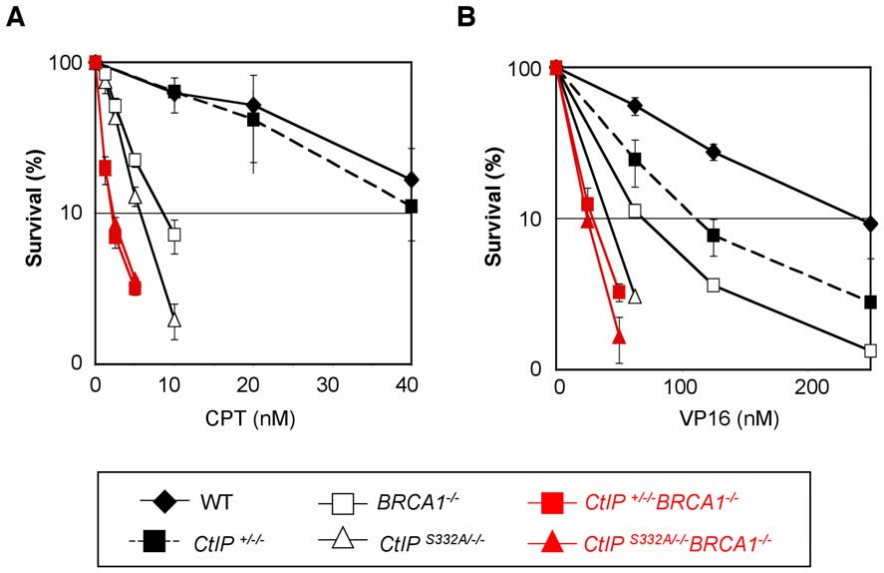
Epistasis analysis of BRCA1 and CtIP in cellular tolerance to topoisomerase inhibitors. Cellular survival of each genotype was assessed by measuring the amount of ATP in cell lysates after 48-h exposure to CPT (A) or VP16 (B). The survival curves for the *CtIP^S332A/−/−^BRCA1^−/−^* cells and their control *CtIP^+/−/−^BRCA1^−/−^* cells are depicted in red. At least five independent experiments were performed with triplicate samples, and the representative results are shown. The error bars indicate S.D.

We next examined the sensitivity of double mutant cells to CPT and VP16. To this end, we measured the number of live cells after 48-hour continuous exposure to the DNA-damaging agents [32], during which the double mutant cells are able to divide four to five times. We did not use a conventional colony formation assay for this purpose, because *CtIP^+/−/−^BRCA1^−/−^* and *CtIP^S332A/−/−^BRCA1^−/−^* clones grew very badly from a single cell in semi-solid methylcellulose medium. The number of viable cells cultured in the presence of CPT was significantly decreased for *CtIP^S332A/−/−^* and *BRCA1^−/−^* cells compared to the *wild-type* cells, whereas *CtIP^+/−/−^* cells grew to the similar extent to the *wild-type* cells in the presence of CPT (Figure 6A). The sensitivity of *CtIP^+/−/−^BRCA1^−/−^* cells to CPT was greater than that of *BRCA1^−/−^* clones. This observation is in agreement with the idea that BRCA1 and CtIP can independently contribute to HR, where CtIP promotes the resection of DSBs, while BRCA1 subsequently loads Rad51 at resected ssDNA overhang. Importantly, although the *CtIP^S332A^* mutation significantly increased cellular sensitivity to CPT in the presence of BRCA1, the *CtIP^S332A/−/−^BRCA1^−/−^* and *CtIP^+/−/−^BRCA1^−/−^* clones exhibited a very similar sensitivity to CPT (Figure 6A). Likewise, the *CtIP^S332A/−/−^BRCA1^−/−^* and *CtIP^+/−/−^BRCA1^−/−^* clones exhibited indistinguishable cellular sensitivities to VP16 (Figure 6B). These observations suggest that CtIP and BRCA1 can act in collaboration to repair DSBs that are chemically modified by topoisomerases.

## Discussion

We here show that conditional depletion of CtIP protein led to cellular lethality with increased frequency of chromosomal aberrations in DT40 cells. CtIP depletion abolished the accumulation of RPA and Rad51 at DNA damaged sites, suggesting that it is required for the resection of DSBs during HR, and that this function is essential for the proliferation of cells. These results are in agreement with previous reports [3]. In contrast, the DT40 cells harboring S332A mutation in CtIP showed the accumulation of RPA and Rad51 upon DNA damage, and were able to proliferate with normal kinetics. Remarkably, compared to the *CtIP^+/−/^* ^−^ cells, the *CtIP^S332A/−/−^* clones exhibited significantly increased sensitivity to CPT and VP16, both of which stabilize the Topo-DNA cleavage complex. These observations support the proposition that, in additon to the resection of DSBs, CtIP has the second function, most likely the removal of covalently-bound polypeptides from DSBs. Hence, *CtIP^S332A/−/−^* clones are the novel separation-of-function mutants where CtIP-dependent resection is proficient, whereas the second function required for the tolerance to topoisomerase inhibitors is deficient.

In this study, we demonstrated that the inactivation of *CtIP* in DT40 cells results in cellular death. We speculate that the defective DSB repair during S phase is the primary cause of cellular death rather than the misregulation of RB/E2F pathway [33],[34]. It has been reported that CtIP promotes G1/S progression by releasing RB-imposed repression and by upregulating the genes required for S phase entry such as *cyclin D1*. MEF from *CtIP*-deficient mice and NIH3T3 cells transfected with *CtIP* siRNA arrest at G_1_ phase of cell cycle. In contrast, DT40 cells that are depleted of *CtIP* showed a marked reduction in S phase and an increase in sub-G_1_ population with the spontaneous chromosomal aberrations. We speculate that DT40 cells have a lower threshold to enter the S phase in the presence of DNA damage compared to the other types of cells owing to their character that they lack *p53* expression [35] and overexpress *c-myc* [36].

The phenotype of our *CtIP*-depleted DT40 cells was remarkably different from that of the *CtIP*-deficient DT40 cells generated by Hiom's group [37]. Surprisingly, their *CtIP*-depleted DT40 cells were capable of proliferating. However, we believe that *CtIP* is essential for cellular proliferation because it has been shown that CtIP works together with Mre11/Rad50/Nbs1 complex in budding and fission yeasts as well as in mammalian cells [3],[6],[38], and the increased spontaneous chromosomal aberrations and cellular death observed in our *CtIP*-depleted cells are consistent with our previous reports that deficiency of either one of *Mre11*, *Rad50*, or *Nbs1* was all lethal to DT40 clones [23],[24]. The viability of the *CtIP*-deficient DT40 cells generated by Hiom's group might be due to the occurrence of suppressor mutations during the disruption of the three allelic *CtIP* genes. Another possibility is that the disruption of exons 1 and 2 in Hiom's group might still allow the residual expression of an N-terminal-truncated CtIP protein, as is observed for the expression of an N-terminal-truncated Nbs1 protein in patients with Nijmegen syndrome [39].

Another critically different point between our study and Hiom's group is that they conclude that the phosphorylation of CtIP-S332 promotes the resection of DSBs, whereas our data do not support this conclusion. The discrepancy between the two studies may be attributable to the different ways of introducing the S332A mutation into the DT40 cells. They randomly integrated *wild-type* and *CtIP^S332A^* transgenes at different loci in their “*CtIP*-null” cells, while we inserted the S332A mutant into one of the *CtIP* allelic genes. This knock-in approach is essential for the accurate quantitative evaluation of HR and NHEJ, because the endogenous promoter expresses *CtIP* transcripts differently in each phase of the cell cycle, and this differential expression accounts for the reduced usage of HR in the G_1_ phase in fission yeast [38]. Alternatively, the difference between our results could be because Hiom's group re-introduced human *CtIP* cDNA (wild type or mutants) instead of that derived from chicken into DT40 cells to create individual clones. The human protein may act differently or incompletely in chicken DT40 cells.

The exact function of BRCA1 in HR is controversial. The discovery of the BRCA1-CtIP interaction has led to a proposal that BRCA1 might facilitate the resection step of HR [11],[37],[40]. However, RPA foci are not completely abolished in BRCA1 mutant cells in these reports, suggesting that ssDNA does form in the absence of functional BRCA1. We found that RPA accumulated at the sites of laser microirradiation in *BRCA1^−/−^* and *CtIP^S332A/−/−^* cells, while Rad51 focus formation is impaired in *BRCA1^−/−^* cells. These results indicate that the BRCA1-CtIP interaction is not involved in the promotion of HR including the resection step, and are in agreement with the idea that BRCA1 facilitates the loading of Rad51 on resected ssDNA as does BRCA2 [1],[29],[41]. Recently, it was found that BRCA1 forms a complex with BRCA2 [42], further supporting the collaborative and overlapping function of BRCA1 and BRCA2. Although we cannot formally exclude the possibility that the RPA accumulation is delayed in *BRCA1^−/−^* cells (the extent of RPA accumulation induced by laser irradiation cannot be quantified, and we failed to induce RPA foci by other genotoxic stimuli in DT40 cells), our data, together with the fact that BRCA1 deficiency does not lead to cellular lethality in DT40 cells, indicate that BRCA1 has only a minor role, if any, in the resection step. The discrepancies among researchers may arise from different experimental settings including how BRCA1 is inactivated (by gene targeting, siRNA knockdown, or C-terminal truncation), the cell cycle distribution of each cell type, and the extent of DSB end modifications induced by laser or γ-ray irradiation. Further studies will clarify the differences among each group.

**Figure 7:**
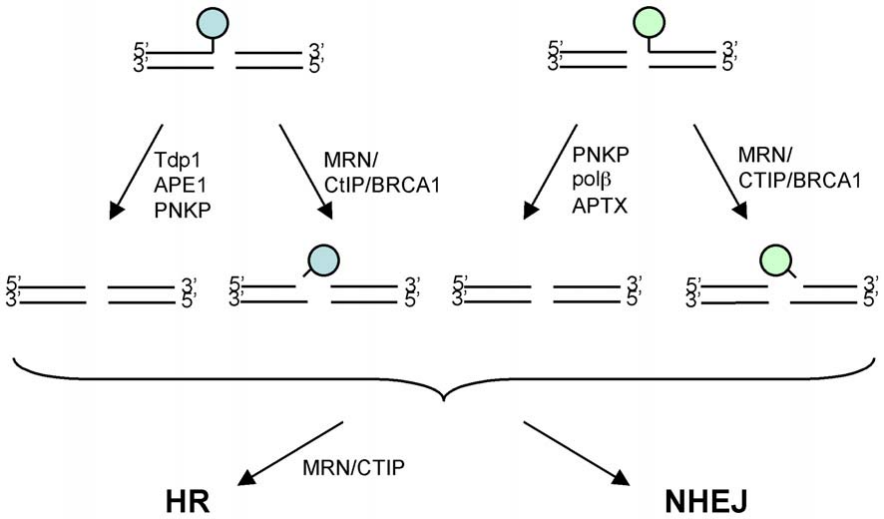
Model for processing the modified DNA ends. Topoisomerase inhibitors induce chemical modification (indicated by a circle) at 3′ and 5′ ends of DSBs, and thereby inhibit subsequent DSB repair. There are two distinct pathways to eliminate such chemical modifications from DSBs. In the first pathway, tyrosyl-DNA phosphodiesterase1 (Tdp1), polynucleotide kinase 3′-phosphatase (PNKP), and AP endonuclease I (APE1) remove various chemical modifications from the 3′ ends of DSBs, while PNKP, DNA polymerase β (Polβ), and aprataxin (APTX) remove those from the 5′ ends of DSBs (the arrows on the left). In the second pathway, MRN-CtIP-BRCA1 may act as an endonuclease and eliminate oligonucleotides covalently bound to polypeptide (the arrows on the right). The resulting processed DSBs are subject to homologous recombination (HR) and nonhomologous-end-joining (NHEJ)-dependent DSB repair.

Accumulating evidence indicates that there are two parallel pathways to eliminate chemical modifications from single-strand breaks and DSBs (Figure 7). Firstly, tyrosyl-DNA phosphodiesterase1 (Tdp1) removes polypeptides covalently bound at the 3′ end of DSBs [43]. Polynucleotide kinase 3′-phosphatase (PNKP) and AP endonuclease I (APE1) are also involved in this process. Likewise, PNKP, DNA polymerase β, and aprataxin remove aberrant chemical modifications from the 5′ ends of DSBs [44]. These enzymes may be capable of accurately repairing damaged bases at DSBs. On the other hand, the second pathway involves endonucleases and removes damaged bases along with proximal intact oligonucleotides from the 3′ or 5′ ends of DSBs. Our study showed that this pathway could contribute to cellular tolerance to alkylating agents such as MMS as well as to topoisomerase inhibitors.

A well-known precedent involving the second pathway is the Mre11/Rad50/Nbs1-complex-dependent elimination of oligonucleotides as well as the covalently associated topoisomerase-like protein (Spo11) from DSBs during meiotic HR in *S. cerevisiae* [2]. A more recent study of the *S. pombe CtIP* mutant (*ctp1Δ*) showed that the level of Top2 protein covalently bound to DNA in the *ctp1Δ* mutant increased during treatment with TOP-53, one of the VP16 derivatives, suggesting that Ctp1 plays a role in the endonuclease-dependent removal of covalently-bound polypeptides from the 5′ end of DSBs [20]. Our study indicates that this conclusion is also relevant to vertebrate cells although there are significant differences between vertebrate and yeast systems. First, yeast Ctp1 or Sae2 seem to be important only for the removal of the peptide covalently bound to 5′ of DSB ends as demonstrated for DNA damage induced by TOP-53 or Spo11 [2],[20]. Second, yeast does not have BRCA1 counterpart. BRCA1 is involved in degradation of trapped Topo1 cleavage complexes along with proteasome [45]. We hypothesize that BRCA1 may facilitate the removal of Topo1 by degrading them to small polypeptides, which in turn are removed with oligonucleotides by the nuclease activity of CtIP. In summary, we here show compelling evidence that the collaborative action of BRCA1 and CtIP plays a critical role in the endonuclease-dependent removal of damaged nucleotides from DSBs, and acts on the processed DSBs for subsequent HR and NHEJ.

## Materials and Methods

### Cell culture

DT40 cells were cultured in RPMI-1640 medium supplemented with 10^−5^ M β-mercaptoethanol, penicillin, streptomycin, 10% fetal calf serum (FCS), and 1% chicken serum (Sigma, St Louis, MO, USA) at 39.5°C.

### Generation of *CtIP* conditional mutant DT40 cells

To generate *CtIP* gene disruption constructs, genomic DNA sequences of DT40 cells were amplified using primers 5′-GGATGCGGAGAGGCTTGAAGAGTTTTACAC-3′ and 5′-TTACAGCACAACGATCACATAATCCCGCTC-3′ for the 5′ arm, and 5′-GGAGCTTCTAGCAATACGCGGAACAACTCA-3′ and 5′-GCTTCCCCTCCAATTCTTGACTGAGAATCA-3′ for the 3′ arm. The amplified PCR products were cloned into the pCR2.1-TOPO vector (Invitrogen, CA, USA). The *Bam*HI site in the plasmid that contains the 5′ arm was disrupted by blunt-self ligation. The 1.6-kb *Hind*III fragment was ligated into the partially-digested *Hind*III site of the 3.0-kb 3′ arm containing the plasmid. A drug-resistance gene (hisD or bsr) was inserted into the *Bam*HI site of the pCR2.1 vector containing both the 5′ and 3′ arms. To generate *CtIP^+/−/−^* cells, linearized *CtIP* gene-disruption constructs were transfected sequentially by electroporation (BioRad). The genomic DNA of the transfectants was digested with *Sac*I and the targeted clones were confirmed by Southern blot analysis. The 0.5-kb fragment was amplified using primers 5′-GATTGTATGCTTCAGAGGCTCCTGC-3′ and 5′-GAAATTCCCAACTTTAGCTCCCCTTGAC-3′ and used as a probe. To construct the *CtIP* expression plasmid, chicken the *CtIP* open reading frame was amplified by PCR, using the primers 5′-GGGGACAAGTTTGTACAAAAAAGCAGGCTTCGAACCATGAATGCGTCTGGGGGAACTTGTG-3′ and 5′-GGGGACCACTTTGTACAAGAAAGCTGGGTCTTATGTCTTCTGCTCTTTGCCTTTTGG-3′, and cloned into a Gateway donor vector, pDONR207 (Invitrogen, CA, USA), by BP reaction. The *CtIP* gene in the donor vector was transferred to an expression vector (pA-puro) containing the Gateway conversion cassette under the *β-actin* promoter by LR reaction. To construct a *CtIP* expression vector under the control of a tetracycline-repressible promoter, *CtIP* cDNA was amplified by PCR, using the primers 5′-CTCGAGATGAATGCGTCTGGGGGAACTTGTG-3′ and 5′-GTCGACTTATGTCTTCTGCTCTTTGCCTTTTGG-3′, and cloned into pCR2.1-TOPO vector. The *Xho*I-*Sal*I fragment containing the *CtIP* cDNA was blunted and cloned into the *Eco*RV site of a modified pTRE2 vector (Clontech, CA, USA) containing a loxP-flanked puromycin-resistant cassette.

*CtIP^+/−/−^* cells were introduced with the tetracycline-controlled trans-activator (tTA) gene through retrovirus infection. Infected cells were sub-cloned, and tTA expression was confirmed by western blot analysis. The resulting tTA-expressing *CtIP^+/−/−^* cells were transfected with the pTRE2 puro^R^/CtIP, and puromycin-resistant clones were selected to isolate the *CtIP^+/−/−^tetCtIP* cells. The puromycin-resistance gene was then deleted by transiently expressing the Cre recombinase (Amaxa solution T, program B-23). Puromycin-sensitive *CtIP^+/−/−^tetCtIP* cells were transfected with the *CtIP* gene-disruption construct carrying the puromycin-resistant cassette to generate *CtIP^−/−/−^tetCtIP* cells.

### Generation of *CtIP^S332A/−/−^* mutant cells

The targeting vectors for the *CtIP* S332A mutants were generated by site-directed mutagenesis. To generate the S332A knock-in vector, genomic DNA was amplified by PCR, using primers 5′-ATTATGCCCCTGAAAGAAGGGAAAC-3′ and 5′-TTTCCTGGGTTTGCTCTTGATTTT-3′, and cloned into the pCR2.1-TOPO vector (Invitrogen, CA, USA). Site-directed mutagenesis was performed using primers 5′-GATTCTCAGGTAGTTGCTCCTGTTTTCGGA-3′ and 5′-TCCGAAAACAGGAGCAACTACCTGAGAATC-3′. The puromycin-resistance gene was inserted into the *Hpa*I site of the resulting plasmid. After transfection of the S332A knock-in vector into the *CtIP^+/+/−^* cells, the targeted clones were selected against puromycin and then identified by Southern blot analysis of genomic DNA digested with *Hind*III. To make probe DNAs, the 0.6-kb fragments were amplified using primers 5′-GACTAACAAAGATCAACCTGTC-3′ and 5′-GTGCATGAGATTTTGGTCGTTG-3′. After the deletion of the puromycin-resistance gene by transiently expressing Cre recombinase by nucleofection (Amaxa, Germany), the third allele of the *CtIP* gene was disrupted by transfecting the *CtIP* gene-disruption construct carrying the puromycin-resistance gene. The insertion of the S332A mutation into the endogenous *CtIP* gene was confirmed by RT-PCR followed by sequencing amplified DNA.

### Generation of *CtIP^+/−/−^BRCA1^−/−^* and *CtIP^S332A/−/−^BRCA1^−/−^* clones

The puromycin-resistant cassette in the targeting vector for the *BRCA1* gene [31] was replaced with the neomycin-resistant cassette. *CtIP^+/−/−^* and *CtIP^S332A/−/−^* cells were sequentially transfected with targeting vectors containing the puromycin- and neomycin-resistant gene, and selected against G418 and puromycin, respectively. The clones with the disrupted *BRCA1* gene were identified by Southern blot analysis as described previously [31].

### Real-time PCR quantification of gene expression

Quantitative real-time PCR was performed in an ABI Prism 7000 sequence detector (Applied Biosystems) using SYBR Green PCR Master Mix reagent (Applied Biosystems) according to the manufacturer's instruction. *CtIP* cDNA was amplified using primers 5′-GGAATTGGAGGAGCAAAAGCAAC-3′ and 5′-GAAACTCACTGTTGCTCTTTG-3′. The expression level of *CtIP* was normalized against *β-actin* using the comparative CT method.

### Western blotting analysis

For Western blot analysis, the antibodies specific for CtIP (BL1914, Bethyl, TX, USA), β-actin (Sigma, MO, USA), Rad51 (Ab-1, Calbiochem, CA, USA) were used for detection of each protein. Secondary antibodies were horseradish peroxidase (HRP)-conjugated antibodies to mouse Ig (GE Healthcare, MA, USA) and HRP-conjugated antibody to rabbit Ig (Santa Cruz, CA, USA).

### Chromosome aberration analysis

Karyotype analysis was performed as described previously [25]. To measure the number of γ-ray-induced chromosome breaks in mitotic cells, we exposed cells to 2 Gy γ-rays and immediately added colcemid. At 3 hours after irradiation, mitotic cells were harvested and subjected to chromosome analysis.

### Measurement of cellular sensitivity to DNA–damaging agents

Methylcellulose colony formation assays were performed as described previously [30],[46]. Since in this assay the plating efficiency of *BRCA1*-deficient cells was less than 50%, we used a different assay to measure cellular sensitivity to DNA-damaging agents. Cells (1×10^3^) were seeded into 24-well plates containing 1 ml culture medium per well and the DNA-damaging agents, and then incubated at 39.5°C for 48 hours. To assess the number of live cells, we measured the amount of ATP in the cellular lysates. We confirmed that the number of live cells was closely correlated with the amount of ATP. This ATP assay was carried out with 96-well plates using a CellTiter-Glo Luminescent Cell Viability Assay Kit (Promega Corporation, WI, USA). Briefly, we transferred 100 µl of cell suspension to the individual wells of the plates, placed the plates at room temperature for approximately 30 minutes, added 100 µl of CellTiter-Glo Reagent, and mixed the contents for 2 minutes on an orbital shaker to induce cell lysis. The plate was then incubated at room temperature for 10 minutes to stabilize the luminescent signal. Luminescence was measured by Fluoroskan Ascent FL (Thermo Fisher Scientific Inc., MA, USA).

### I-*Sce*-I–induced gene conversion and targeted integration frequencies

The measurement of homologous recombination frequencies using a *SCneo* cassette [28] and *CENP-H-EGFP* was performed as described previously [47]. After the I-*Sce*-I vector was transfected into the cells, the frequency of neomycin-resistant colony formation was measured.

### Synchronization of cells

To enrich DT40 cells in the G_1_ phase, cells were synchronized by centrifugal counterflow elutriation (Hitachi Industrial, Japan). The cell suspension (∼5×10^7^) was loaded at a flow rate of 11 ml/min into an elutriation chamber running at 2,000 rpm. The first 50 ml was discarded, and the following 100 ml was used as a G_1_-phase cell fraction.

### Microscopy imaging and generation of DNA damage

Fluorescence microscopy was carried out and images were obtained and processed using the IX81 (Olympus, Japan). Cells were cultured in medium containing BrdU (10 µM) for 24–48 h to sensitize them to DSB generation by means of a 405 nm laser from a confocal microscope (FV-1000, Olympus, Japan). During laser treatment, cells were incubated in phenol red-free Opti medium (GIBCO, NY, USA) to prevent the absorption of the laser's wavelength. γ-irradiation was performed using ^137^C (Gammacell 40, Nordion, Kanata, Ontario, Canada). Antibodies against Rad51 (Ab-1, Calbiochem, CA, USA), FK2 (Nippon Biotest Laboratories, Japan), RPA p32 (GeneTex, TX, USA), rabbit Ig (Alexa 488-conjugated antibody, Molecular Probe, OR, USA), and mouse Ig (Alexa 594-conjugated antibody, Molecular probe, OR, USA) were used for visualization.

## Supporting Information

Figure S1 Generation of *CtIP^−/−/−^tetCtIP* mutants. (A) *CtIP* gene disruption strategy. The map shows the organization of the *CtIP* gene (top), targeting construct (middle), and targeted allele (bottom). Black and white boxes represent exons and the drug-marker cassettes, respectively. (B) Genomic PCR analysis at the disrupted site using primers p1 and p2, as shown in (A). (C) Southern blot analysis of *Sac*I-digested genomic DNA using the probe shown in (A).
(11.61 MB TIF)

Figure S2 Generation of *CtIP^S332A/−/−^* mutants. (A) The strategy for the generation of *CtIP^S332A/−/−^* mutants. The knock-in vectors shown in Figure S3B were introduced into the *CtIP^+/+/−^* cells. The insertion of the S332A mutation was verified by Southern blot analysis of *Hind*III-digested genomic DNA. Cre recombinase were transiently expressed in the resulting *CtIP^S332A/+/−^* clones to delete the drug-resistant marker. The remaining intact *CtIP* allele was targeted by the *CtIP* disruption construct to obtain *CtIP^S332A/−/−^* clones. (B) The knock-in constructs for the generation of *CtIP^S332A/−/−^* mutant clones. Black and white boxes represent exons and the drug-marker cassettes, respectively. (C) Nucleotide sequence analysis of *CtIP* cDNAs derived from the *CtIP^+/−/−^* and *CtIP^S332A/−/−^* mutant. The total RNA was subjected to reverse transcription. The regions spanning the mutations were amplified by PCR and the sequence was determined. (D) Cell cycle profiles of *CtIP^S332A/−/−^* and *CtIP^F871A/−/−^* mutant cells. Cells were pulse-labeled with BrdU for 10 min and subsequently stained with FITC-conjugated anti-BrdU antibody (Y axis, log scale) and propidium iodide (PI) (X axis, linear scale). (E) Western blot analysis of *wild-type*, *CtIP^+/−/^* ^−^ and *CtIP^S332A/−/−^* DT40 clones. β-actin was used as a loading control. (F) FLAG-BRCA1 association with CtIP is dependent on Ser332. 293T cells were transfected with plasmids encoding FLAG-tagged chicken BRCA1 together with either *wild-type* CtIP or S332A CtIP. Cell lysates were subjected to immuno-precipitation with anti-FLAG antibody, and the precipitated proteins were detected with anti-FLAG or anti-CtIP antibody. (G) Quantitative real time PCR of *CtIP* mRNA in *wild-type*, *CtIP^+/−/^* ^−^ and *CtIP^S332A/−/−^* DT40 clones. PCR amplification was performed in triplicate. The expression level of *CtIP* was normalized against *β-actin* using the comparative CT method.
(19.89 MB TIF)

Figure S3 Sensitivity of *CtIP^S332A/−/−^* mutant to IR at G_1_ phase. Cells at G**_1_** phase were separated by centrifugal elutriation and were γ-irradiated for colony survival assay. The dose of γ-ray irradiation is displayed on the X axis on a linear scale, while the percent fraction of surviving colonies is displayed on the Y axis on a logarithmic scale.
(10.15 MB TIF)

Figure S4 Generation of *CtIP^+/−/−^BRCA1^−/−^* and *CtIP^S332A/−/−^BRCA1^−/−^* mutants. (A) Southern blot analysis of double mutant clones. The genomic DNA of indicated genotype was digested with *Hind*III and hybridized with the probe which detects the 3′ of the targeted *BRCA1* site. The top band at 10 kb and the bottom band at 4.5 kb correspond to the *BRCA1* allele disrupted with neomycin (NEO)- and puromycin (Puro)-resistance cassette, respectively. The middle band at 5.7 kb is the non-targeted internal allele of *BRCA1* gene. (B) RT–PCR of double mutant clones. cDNA was synthesized from each genotype and was used for PCR amplification of *BRCA1* (upper panel) or *β-actin* (lower panel) as a control.
(7.59 MB TIF)
