# Supplementary figures and images for: Collaborative Action of Brca1 and CtIP in Elimination of Covalent Modifications from Double-Strand Breaks to Facilitate Subsequent Break Repair
Source: PLoS Genet. 2010 Jan 22;6(1):e1000828. doi: 10.1371/journal.pgen.1000828 (PMC2809774; doi:10.1371/journal.pgen.1000828)

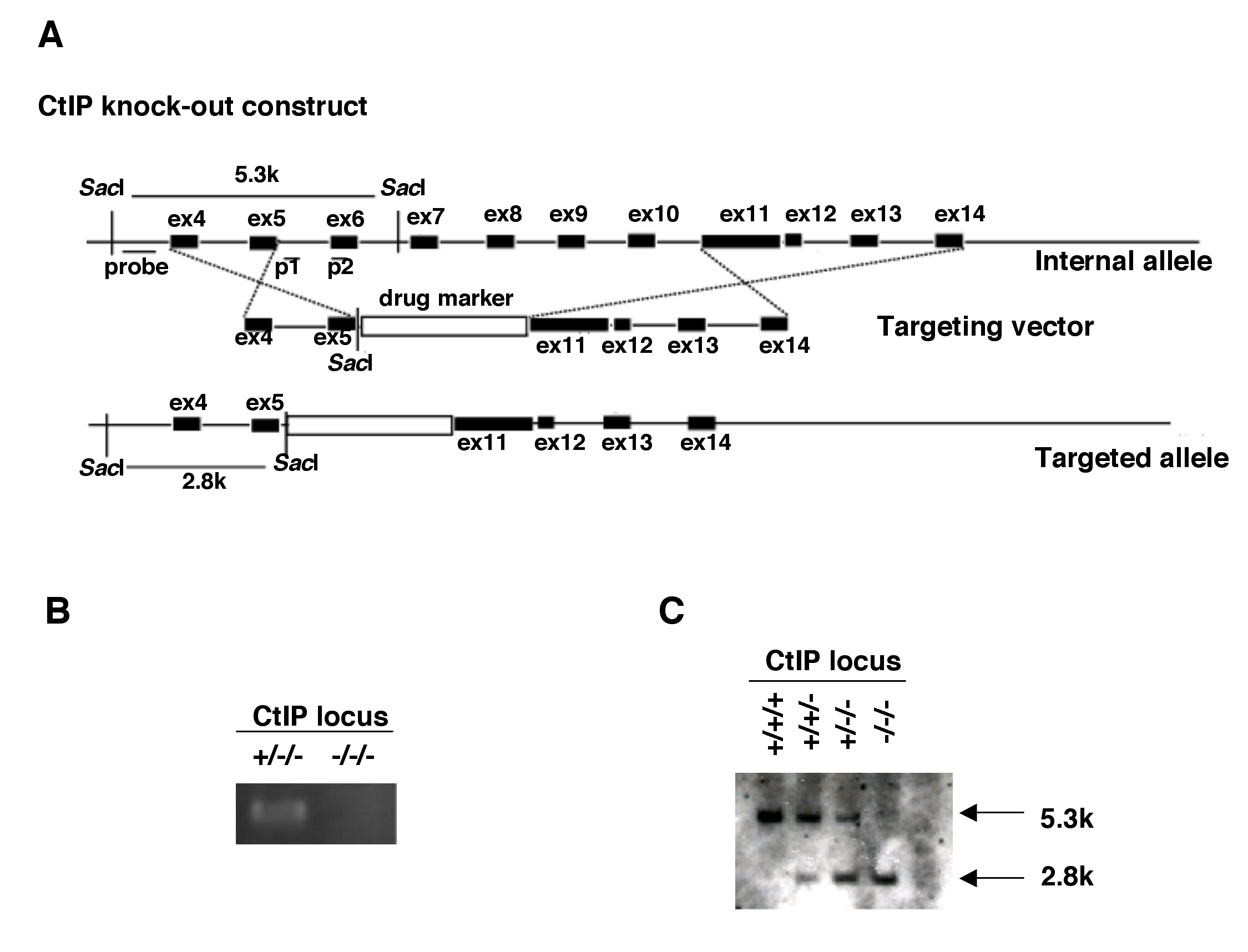

Supplement: Figure S1 — Generation of CtIP−/−/−tetCtIP mutants. (A) CtIP gene disruption strategy. The map shows the organization of the CtIP gene (top), targeting construct (middle), and targeted allele (bottom). Black and white boxes represent exons and the drug-marker cassettes, respectively. (B) Genomic PCR analysis at the disrupted site using primers p1 and p2, as shown in (A). (C) Southern blot analysis of SacI-digested genomic DNA using the probe shown in (A). (11.61 MB TIF) [file pgen.1000828.s001.tif]

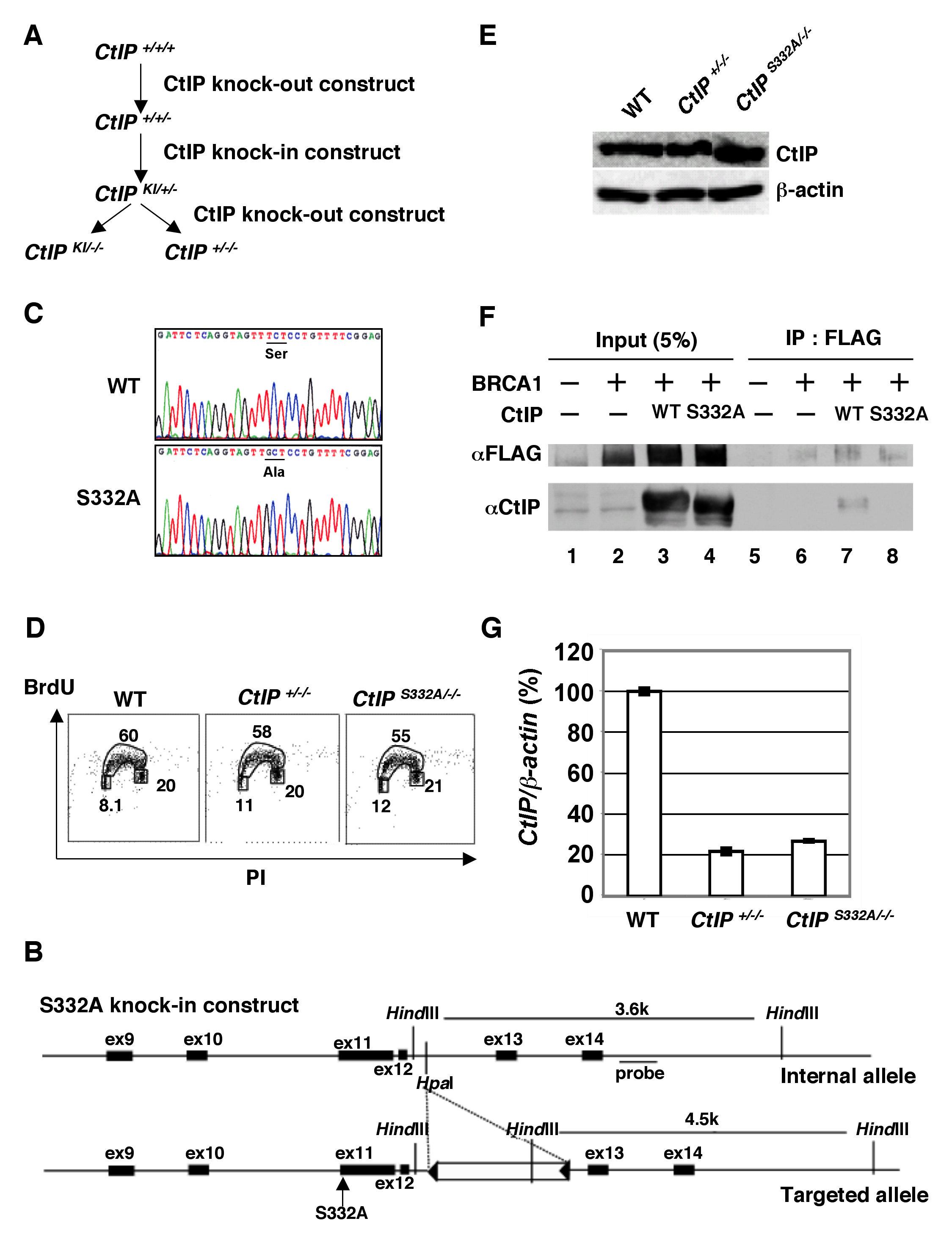

Supplement: Figure S2 — Generation of CtIPS332A/−/− mutants. (A) The strategy for the generation of CtIPS332A/−/− mutants. The knock-in vectors shown in Figure S3B were introduced into the CtIP+/+/− cells. The insertion of the S332A mutation was verified by Southern blot analysis of HindIII-digested genomic DNA. Cre recombinase were transiently expressed in the resulting CtIPS332A/+/− clones to delete the drug-resistant marker. The remaining intact CtIP allele was targeted by the CtIP disruption construct to obtain CtIPS332A/−/− clones. (B) The knock-in constructs for the generation of CtIPS332A/−/− mutant clones. Black and white boxes represent exons and the drug-marker cassettes, respectively. (C) Nucleotide sequence analysis of CtIP cDNAs derived from the CtIP+/−/− and CtIPS332A/−/− mutant. The total RNA was subjected to reverse transcription. The regions spanning the mutations were amplified by PCR and the sequence was determined. (D) Cell cycle profiles of CtIPS332A/−/− and CtIPF871A/−/− mutant cells. Cells were pulse-labeled with BrdU for 10 min and subsequently stained with FITC-conjugated anti-BrdU antibody (Y axis, log scale) and propidium iodide (PI) (X axis, linear scale). (E) Western blot analysis of wild-type, CtIP+/−/ − and CtIPS332A/−/− DT40 clones. β-actin was used as a loading control. (F) FLAG-BRCA1 association with CtIP is dependent on Ser332. 293T cells were transfected with plasmids encoding FLAG-tagged chicken BRCA1 together with either wild-type CtIP or S332A CtIP. Cell lysates were subjected to immuno-precipitation with anti-FLAG antibody, and the precipitated proteins were detected with anti-FLAG or anti-CtIP antibody. (G) Quantitative real time PCR of CtIP mRNA in wild-type, CtIP+/−/ − and CtIPS332A/−/− DT40 clones. PCR amplification was performed in triplicate. The expression level of CtIP was normalized against β-actin using the comparative CT method. (19.89 MB TIF) [file pgen.1000828.s002.tif]

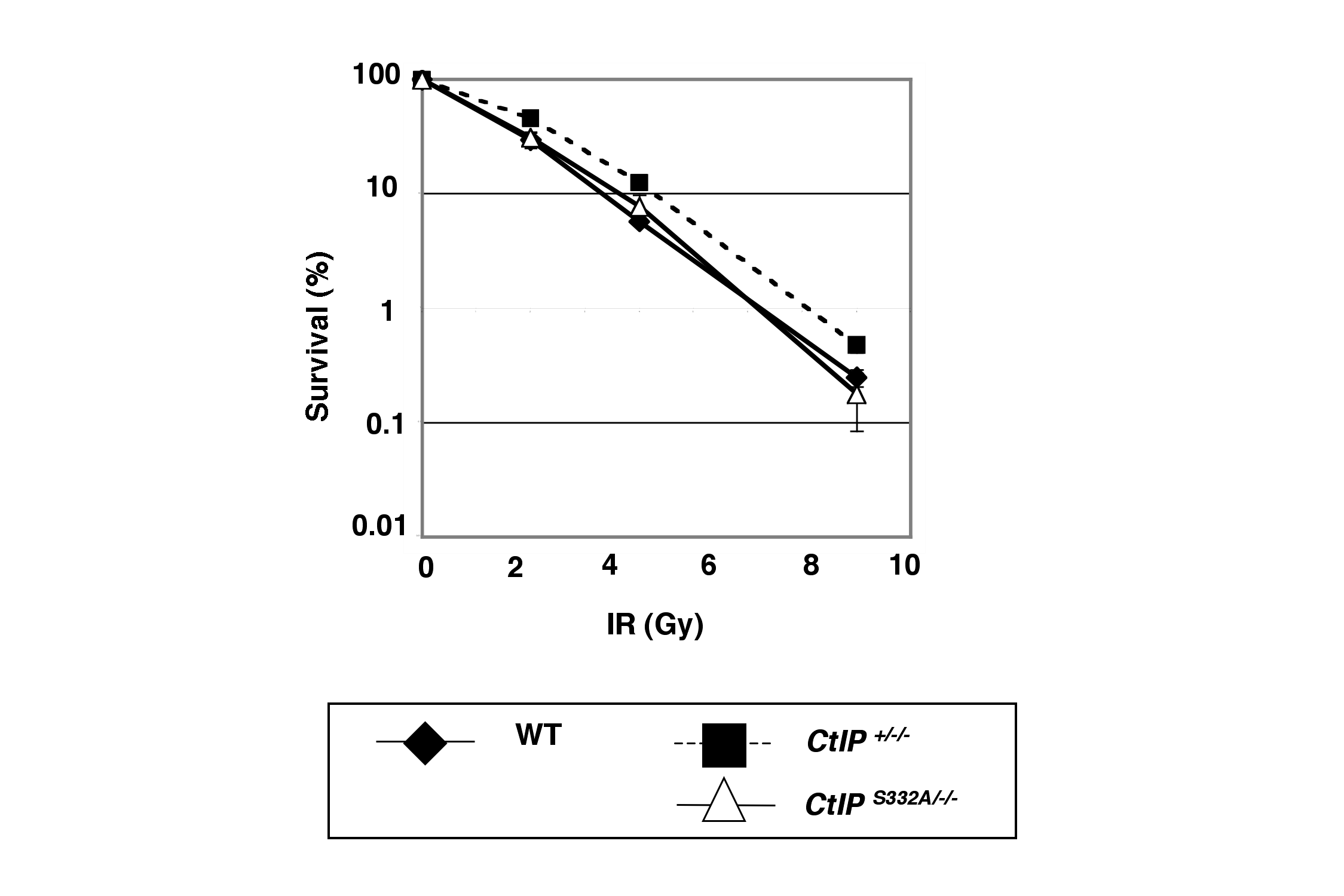

Supplement: Figure S3 — Sensitivity of CtIPS332A/−/− mutant to IR at G1 phase. Cells at G1 phase were separated by centrifugal elutriation and were γ-irradiated for colony survival assay. The dose of γ-ray irradiation is displayed on the X axis on a linear scale, while the percent fraction of surviving colonies is displayed on the Y axis on a logarithmic scale. (10.15 MB TIF) [file pgen.1000828.s003.tif]

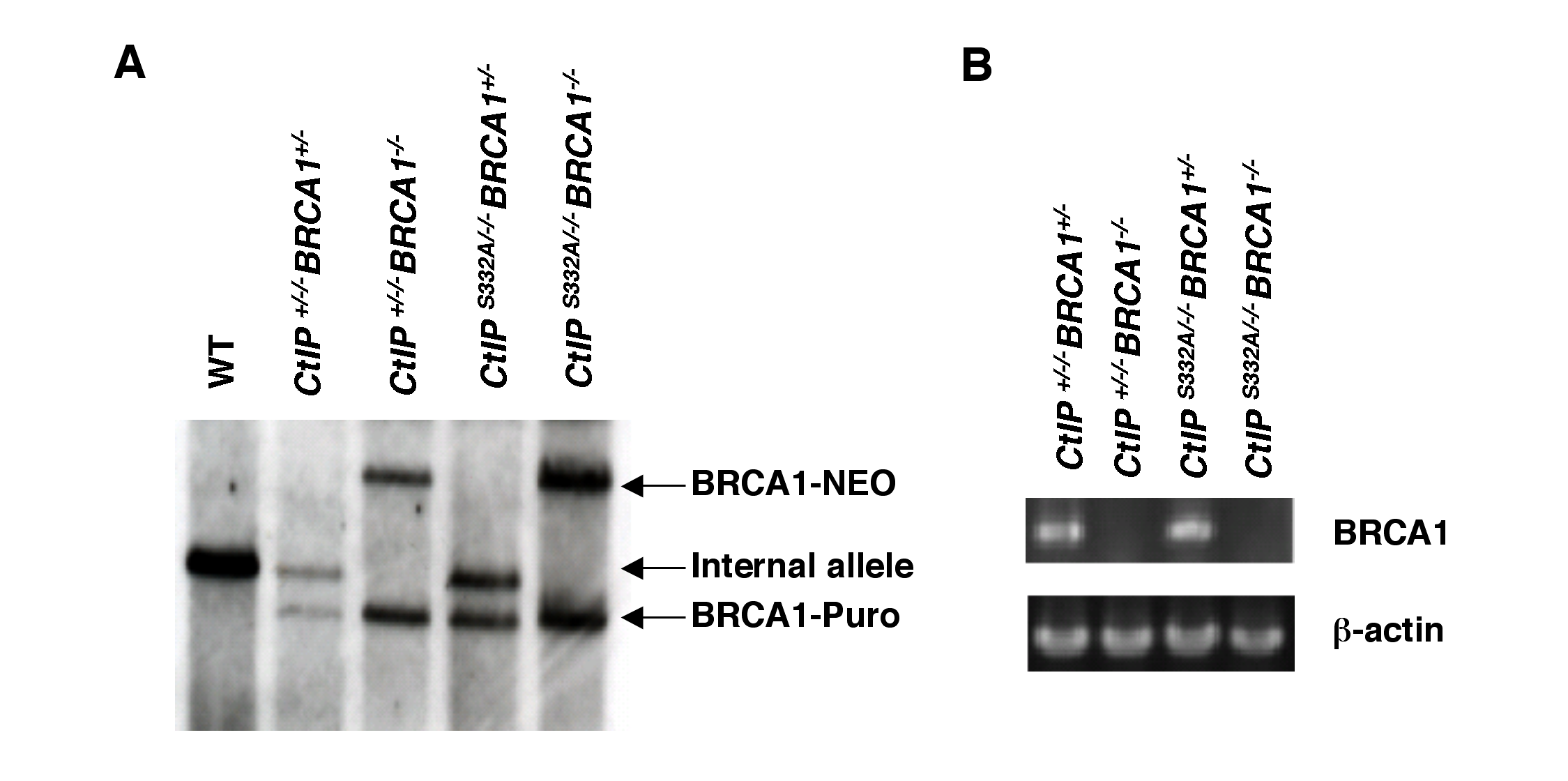

Supplement: Figure S4 — Generation of CtIP+/−/−BRCA1−/− and CtIPS332A/−/−BRCA1−/− mutants. (A) Southern blot analysis of double mutant clones. The genomic DNA of indicated genotype was digested with HindIII and hybridized with the probe which detects the 3′ of the targeted BRCA1 site. The top band at 10 kb and the bottom band at 4.5 kb correspond to the BRCA1 allele disrupted with neomycin (NEO)- and puromycin (Puro)-resistance cassette, respectively. The middle band at 5.7 kb is the non-targeted internal allele of BRCA1 gene. (B) RT–PCR of double mutant clones. cDNA was synthesized from each genotype and was used for PCR amplification of BRCA1 (upper panel) or β-actin (lower panel) as a control. (7.59 MB TIF) [file pgen.1000828.s004.tif]
